# Mycotic Aneurysm: A Rare Etiology of a Common Presentation

**DOI:** 10.7759/cureus.27105

**Published:** 2022-07-21

**Authors:** Avinash Ram, Jonathan Deslouches, Sheena Punnapuzha

**Affiliations:** 1 Emergency Medicine, The Brooklyn Hospital Center, Brooklyn, USA

**Keywords:** mycotic aneurysm, id critical care, critical emergency medicine, cardiothoracic imaging, infectious disease pathology, mycotic aortic aneurysm

## Abstract

Mycotic aneurysms are a rare complication of systemic infections, where the arterial vessel wall becomes dilated secondary to bacterial, fungal, or viral infection. The incidence of mycotic aneurysms is rare but carries a significant mortality risk. Patients with mycotic aneurysms can have wide-ranging clinical presentations depending on the site of the aneurysm. Our case discusses one of the most encountered emergency department (ED) presentations, i.e., nausea and vomiting, as a presenting symptom of a patient found to have a mycotic aneurysm.

A 56-year-old patient with a history of HIV, unknown viral load or CD4 count, and admitted IV drug use presented to the ED by ambulance with multiple episodes of nausea and non-bloody vomiting. The patient was noted to be afebrile but met systemic inflammatory response syndrome (SIRS) criteria, necessitating a further workup. CT of the abdomen and pelvis was notable for a saccular aneurysm involving the infrarenal aorta with a large thrombosed component.

This case highlights the importance of early consideration of infected (mycotic) aneurysms in the appropriate patient setting, as delayed diagnosis increases the risk of rupture and death. In a case of non-specific nausea and vomiting, it is not unreasonable to assume this presentation could be attributed to a more benign process, delaying the diagnosis. It may, therefore, be prudent for emergency service providers to add mycotic aneurysms to the differential diagnosis for patients with appropriate risk factors, as presentations of mycotic aneurysms vary greatly.

## Introduction

Mycotic aneurysms are a rare complication of systemic infections in which the arterial vessel wall becomes dilated secondary to bacterial, fungal, or viral infection. It is important to recognize this disease process early, as non-treatment can lead to fulminant sepsis, arterial rupture, and death. Approximately 7-24% of infected aortic aneurysms demonstrate free rupture, and a further 47-61% demonstrate contained or impending rupture at presentation [1]. Patients with mycotic aneurysms can have wide-ranging clinical presentations, depending on the aneurysm site, with the most common physical exam finding being a tender indurated abdominal mass and bruit [2]. Reported presenting symptoms range from hemoptysis to GI bleeding to heart failure [3,4]. This case report highlights a common, non-specific emergency department (ED) complaint, i.e., non-specific nausea and vomiting, with the importance of consideration and early recognition of mycotic aneurysms.

## Case presentation

A 56-year-old male with a history of HIV, unknown viral load, and admitted IV drug use presented to the ED by ambulance with multiple episodes of nausea and vomiting. On arrival, his vitals were notable for a respiratory rate (RR) of 34 per minute and heart rate (HR) of 126 beats per minute. The rest of the vitals including temperature, blood pressure, and oxygen saturation were within normal limits. Physical examination was notable for tenderness to the epigastric region. An electrocardiogram (ECG) showed sinus tachycardia. Chest X-ray showed bilateral opacities, consistent with multifocal pneumonia. Pertinent laboratory values returned as noted in Table 1.

**Table 1:** Pertinent laboratory values

Lab	Results
White blood cell count	10.8 K/cmm (4.8-10.8 K/cmm)
% Neutrophils	92.6% (42.2-75.2%)
Lactic acid	3.5 mmol/L (0.5-2.2 mmol/L)
Troponin	1.17 ng/mL (<0.03 ng/mL)
D-dimer	>4.4 mg/L (0.5 mg/L)

**Figure 1:**
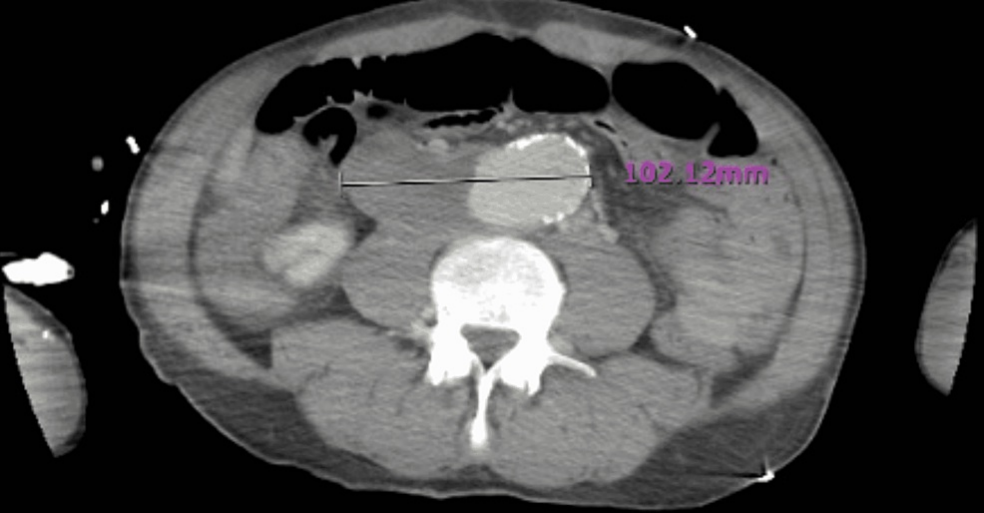
CT scan showing saccular aneurysm with thrombosed component

While in the ED, the patient began to deteriorate clinically, with a RR of 50 per minute and HR of 215 beats per minute. CT of the abdomen and pelvis was performed and showed a saccular aneurysm involving the infrarenal aorta with a large thrombosed component. The aneurysm measured 5 cm in length by 10.2 cm transverse by 3.6 cm anteroposterior (Figure 1).

The patient was admitted to the intensive care unit where blood cultures that were drawn in the ED for the sepsis workup returned positive for *Staphylococcus aureus*. The patient was subsequently transferred to another facility for cardiothoracic surgery evaluation and management.

## Discussion

Non-specific gastrointestinal complaints are one of the leading causes of emergency room visits each year, making up about 5-10% of all United States ED visits annually [5]. With a wide range of possible etiologies, a proper history and physical examination are helpful in narrowing down a diagnosis. Ultimately, however, imaging is often required to make a specific diagnosis. Mycotic aneurysm manifestations are non-specific and can easily be missed. Populations at risk for mycotic aneurysms include IV drug users, immunocompromised hosts, and patients who are status post invasive intravascular procedures [6]. Physical exam findings can vary, as these findings are dependent on the location of the aneurysm. However, the most common finding encountered in these patients is a tender indurated abdominal mass and bruit [2]. Labs are non-specific for the identification of these infected aneurysms. White blood cell count and inflammatory markers are usually elevated in infected aneurysms, but they are non-specific. Blood cultures, while important to obtain, take time to come back and do not add information in the early stages of the diagnosis. Ultimately, radiologic findings are the most sensitive and specific indicators for infected aneurysms, with the imaging modality of choice being CT angiography (CTA) [7]. Findings on CTA that are highly suggestive of mycotic aneurysm include saccular aneurysm with lobulated contours, soft tissue inflammation surrounding a vessel, intramural air around the blood vessel, and perianeurysmal fluid collection [8]. Our patient presented with non-specific nausea and vomiting. As there was no reported abdominal pain, it is not unreasonable to assume that there might be instances where this presentation could be attributed to a more benign process, further delaying the diagnosis and increasing mortality risk. Ultimately, the CT scan was able to provide the definitive diagnosis. However, the main barrier to diagnosis is not a lack of a CT scanner, but a low index of suspicion. This case highlights the importance of developing a broad differential diagnosis, early consideration, recognition, and treatment of infected (mycotic) aneurysms.

## Conclusions

Our patient presented with nausea and vomiting, one of the most commonly encountered ED visit complaints. Given the unremarkable physical exam, this presentation could easily be attributed to a more benign process, delaying the diagnosis and increasing mortality risk. This case highlights the importance of early consideration of infected (mycotic) aneurysms in patients with the appropriate risk factors, as presentations vary greatly.

## References

[1] Brown SL, Busuttil RW, Baker JD, Machleder HI, Moore WS, Barker WF (1984). Bacteriologic and surgical determinants of survival in patients with mycotic aneurysms. J Vasc Surg.

[2] Johnson JR, Ledgerwood AM, Lucas CE (1983). Mycotic aneurysm. New concepts in therapy. Arch Surg.

[3] Shiraishi M, Ohki S, Misawa Y (2011). Mycotic superior mesenteric pseudoaneurysm draining into a vein. Interact Cardiovasc Thorac Surg.

[4] Zhao J (2008). Massive upper gastrointestinal bleeding due to a ruptured superior mesenteric artery aneurysm duodenum fistula. J Vasc Surg.

[5] Kamin RA, Nowicki TA, Courtney DS, Powers RD (2003). Pearls and pitfalls in the emergency department evaluation of abdominal pain. Emerg Med Clin North Am.

[6] Tsao JW, Marder SR, Goldstone J, Bloom AI (2002). Presentation, diagnosis, and management of arterial mycotic pseudoaneurysms in injection drug users. Ann Vasc Surg.

[7] Vogelzang RL, Sohaey R (1988). Infected aortic aneurysms: CT appearance. J Comput Assist Tomogr.

[8] Azizi L, Henon A, Belkacem A, Monnier-Cholley L, Tubiana JM, Arrivé L (2004). Infected aortic aneurysms: CT features. Abdom Imaging.

